# Differential Sensitivity of Bat Cells to Infection by Enveloped RNA Viruses: Coronaviruses, Paramyxoviruses, Filoviruses, and Influenza Viruses

**DOI:** 10.1371/journal.pone.0072942

**Published:** 2013-08-30

**Authors:** Markus Hoffmann, Marcel Alexander Müller, Jan Felix Drexler, Jörg Glende, Meike Erdt, Tim Gützkow, Christoph Losemann, Tabea Binger, Hongkui Deng, Christel Schwegmann-Weßels, Karl-Heinz Esser, Christian Drosten, Georg Herrler

**Affiliations:** 1 Institute of Virology, University of Veterinary Medicine Hannover, Hannover, Germany; 2 Institute of Virology, University of Bonn Medical Centre, Bonn, Germany; 3 Institute of Zoology, University of Veterinary Medicine Hannover, Hannover, Germany; 4 Department of Cell Biology and Genetics, College of Life Sciences, Peking University, Beijing, P. R. China; Kantonal Hospital St. Gallen, Switzerland

## Abstract

Bats (*Chiroptera*) host major human pathogenic viruses including corona-, paramyxo, rhabdo- and filoviruses. We analyzed six different cell lines from either *Yinpterochiroptera* (including African flying foxes and a rhinolophid bat) or *Yangochiroptera* (genera *Carollia* and *Tadarida*) for susceptibility to infection by different enveloped RNA viruses. None of the cells were sensitive to infection by transmissible gastroenteritis virus (TGEV), a porcine coronavirus, or to infection mediated by the Spike (S) protein of SARS-coronavirus (SARS-CoV) incorporated into pseudotypes based on vesicular stomatitis virus (VSV). The resistance to infection was overcome if cells were transfected to express the respective cellular receptor, porcine aminopeptidase N for TGEV or angiotensin-converting enzyme 2 for SARS-CoV. VSV pseudotypes containing the S proteins of two bat SARS-related CoV (Bg08 and Rp3) were unable to infect any of the six tested bat cell lines. By contrast, viral pseudotypes containing the surface protein GP of Marburg virus from the family *Filoviridae* infected all six cell lines though at different efficiency. Notably, all cells were sensitive to infection by two paramyxoviruses (Sendai virus and bovine respiratory syncytial virus) and three influenza viruses from different subtypes. These results indicate that bat cells are more resistant to infection by coronaviruses than to infection by paramyxoviruses, filoviruses and influenza viruses. Furthermore, these results show a receptor-dependent restriction of the infection of bat cells by CoV. The implications for the isolation of coronaviruses from bats are discussed.

## Introduction

Coronaviruses (order *Nidovirales*, family *Coronaviridae*) are enveloped positive-stranded RNA viruses that may be pathogenic for mammals and birds. According to a proposal to the International Committee of Taxonomy of Viruses (ICTV) this group of viruses is classified into three genera (*Alpha-, Beta-, and Gammacoronavirus*) [1]. *A* novel genus, *Deltacoronavirus*, has recently been accepted [2]. Alpha- and betacoronaviruses infect mammals, whereas gamma- and deltacoronaviruses have been detected first and foremost in birds. In the search for the origin of the severe acute respiratory syndrome (SARS) coronavirus (SARS-CoV), fecal samples of many different bats were found to contain coronaviral genomic RNA [3]–[9]. Bats have been hypothesized to act as the principal reservoir hosts for alpha- and betacoronaviruses [8], [10]. Sequence analysis suggested that coronaviruses have succeeded to cross the species barrier to different mammalian species several times so that for example the different human coronaviruses OC43, 229E, SARS-CoV and the recently identified MERS-CoV (formerly designated HCoV-EMC) are the result of distinct interspecies transmission events that may be separated from each other by hundreds of years [11], [12]. An enigma in the analysis of the spread of coronaviruses from its reservoir host to other species is the failure of all attempts so far to isolate an infectious virus from bats [3], [4], [6], [7], [11], [13]–[17]. The reason for this is not clear. However, the surface protein Spike (S) appears to be responsible at least for the inability of bat coronaviruses to replicate in non-bat cells. A synthetic recombinant bat SARS-related coronavirus (SARSr-CoV) was able to infect primate or murine cells expressing the receptor for SARS-CoV, human angiotensin-converting enzyme 2 (hACE2) provided that the receptor binding domain in the bat S protein was replaced by that of the S protein of SARS-CoV [18].

The S protein is the largest glycoprotein of coronaviruses projecting from the viral envelope into the environmental space [19]. Thus, it is responsible not only for the corona-like appearance of the surface projections when viewed under the electron microscope but also for the initial interaction of the virus with target cells. The S protein mediates the binding to the cellular receptor. Apart from the above mentioned hACE2 [20], [21], other proteins that have been identified as receptors for coronaviruses are aminopeptidase N [22], [23] for the alphacoronaviruses transmissible gastroenteritis virus (TGEV), feline enteric coronavirus (FECV), and HCoV-229E, and the carcinoembryonic antigen-related cell adhesion molecule 1a (CEACAM1a) for mouse hepatitis virus (MHV) [24]. In addition to binding to a defined protein receptor, some coronaviruses have a sialic acid-binding activity. For viruses like TGEV or avian infectious bronchitis virus (IBV) binding to sialylated macromolecules may not be sufficient for initiation of infection, however, it may increase the binding and infection efficiency [25]–[27]. Some coronaviruses like bovine coronavirus (BCoV) resemble influenza C virus by using N-acetyl-9-O-acetyl neuraminic acid (Neu5,9Ac_2_) as a receptor determinant on cell surface macromolecules for binding to and infection of target cells [28], [29]. Furthermore, they contain an acetylesterase activity that releases the 9-O-acetyl group from Neu5,9Ac_2_ and thus is able to inactivate the receptor determinant [30]. This so-called receptor-destroying enzyme may – in analogy to influenza viruses – help to avoid binding events that do not result in infection, e.g. virus aggregation or binding to infected cells, and thus increase the spread of infection in the host.

Following binding to cell surface receptors, coronaviruses enter host cells by a fusion event that is also mediated by the S protein. With some coronaviruses, e.g. IBV and MHV, efficient fusion activity depends on proteolytic cleavage of the S protein into the subunits S1 and S2 by furin-like enzymes [31]. Other coronaviruses, e.g. SARS-CoV, TGEV, and HCoV-229E, contain the S protein on the viral surface in an uncleaved form. However, for these viruses, proteolytic activation may also be required because inhibitors of cathepsin L prevent the S protein from mediating infection [32]. This protease may act on viruses during virus entry, e.g. in the endosomal compartment. Recently, a human airway trypsin-like protease has also been implicated in the entry of SARS-CoV into respiratory epithelial cells [33].

Apart from coronaviruses, bats (order *Chiroptera*) have been shown to host a variety of emerging viruses [34], [35], comprising different viral families like *Orthomyxoviridae* [36]–[38], *Rhabdoviridae* (especially the genus *Lyssavirus*) [39]–[41], *Paramyxoviridae* [42]–[46], *Filoviridae* [47]–[49], and others (see [34] for more information).

In order to isolate infectious coronaviruses from bats it is necessary to use appropriate cells, i.e. cells that are susceptible to infection. To identify such cells we applied a pseudotype approach to analyze the ability of two different S proteins from SARSr-CoV to mediate infection. These two SARSr-CoV termed Bg08 and Rp3 were identified previously by us and others in Europe and China, representing two distinct virus lineages within this CoV species [9], [11]. A major species barrier for infection of bat cells was found to be at the level of cell surface receptors. Whereas bat cells were easily infected by paramyxoviruses and influenza viruses as well as by pseudotypes containing the glycoproteins of Marburg virus, the S proteins of SARS-CoV and TGEV were able to mediate infection only when the respective cellular receptor, human ACE2 or porcine APN, was expressed on the cell surface. Two S proteins of bat coronaviruses were unable to mediate infection of either of the bat cell lines analyzed.

## Results

### Infection of bat cells mediated by the S proteins of SARS-CoV or TGEV

**Figure 1:**
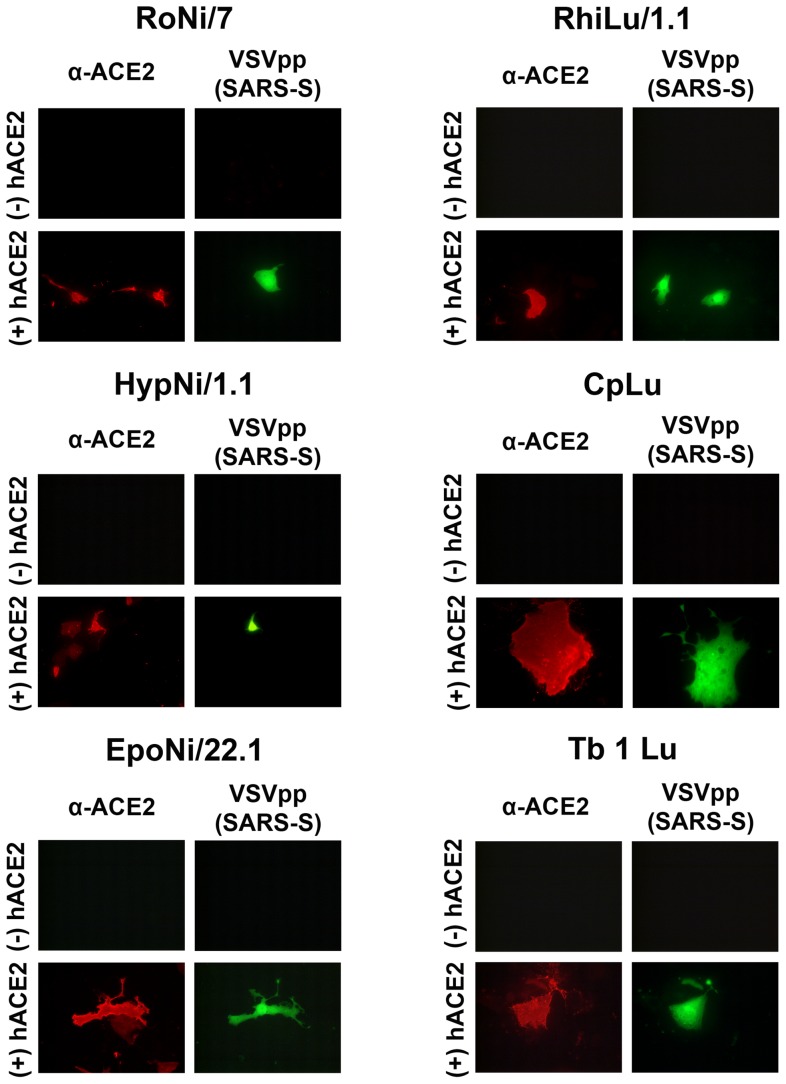
Sensitivity of bat cells to infection by VSV pseudotypes containing the S protein of SARS-CoV. Bat cells (RoNi/7, HypNi/1.1, EpoNi/22.1, RhiLu1.1, CpLu, Tb 1 Lu) were tranfected either with control plasmid (−hACE2) or with an expression plasmid for the human ACE2, the cellular receptor of SARS-CoV (+hACE2). At 24 h post transfection, the cells were infected with VSV pseutotyped with SARS-CoV SΔ18. Expression of hACE2 on the cell surface was detected by antibody staining, whereas VSV pseudotype infection was monitored by EGFP expression. All experiments were performed in triplicates and repeated three times.

**Figure 2:**
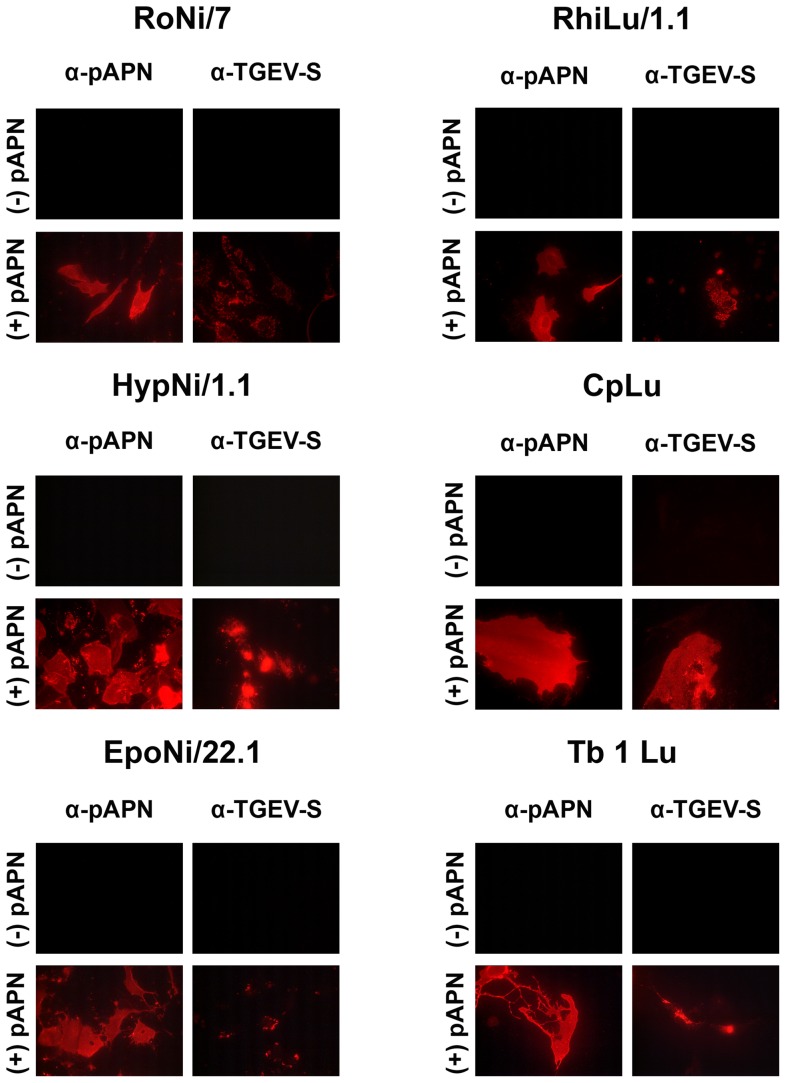
Sensitivity of bat cells to infection by TGEV. Bat cells (RoNi/7, HypNi/1.1, EpoNi/22.1, RhiLu/1.1, CpLu, Tb 1 Lu) were tranfected either with control plasmid (−pAPN) or with an expression plasmid for the porcine APN, the cellular receptor of TGEV (+pAPN). At 24 h post transfection, the cells were infected with TGEV. Expression of pAPN on the cell surface as well as intracellular TGEV antigen was detected by antibody staining. All tests were performed in triplicates and repeated three times.

Infection by coronaviruses is usually restricted to cells of the respective host or cells from related species. A major species barrier is the virus receptor on the surface of the target cell, e.g. hACE2, the receptor for SARS-CoV, and pAPN, the receptor for TGEV. We assessed whether this restriction is also valid for bat cells. For this purpose, a number of bat cells were analyzed whether they are susceptible to infection mediated by the S proteins of SARS-CoV or TGEV. The S protein of SARS-CoV was investigated with the help of the pseudotype system based on vesicular stomatitis virus (VSV). VSV pseudotypes containing SARS-CoV S protein are efficient in infecting Vero E6 cells [50]–[52]. As shown in Figure 1, out of six cell lines derived from the kidney (Ni) or lung (Lu) of *Yinpterochiroptera* (genera, *Rousettus* (Ro), *Hypsignathus* (Hyp), *Epomops* (Epo), or *Rhinolophus* (Rhi)) or *Yangochiroptera* (genera *Carollia* (Cp), *Tadarida* (Tb)), none was susceptible to SARS-CoV S-mediated infection (Figure 1). When the cells were transfeced with a plasmid for expression of hACE2, all cell lines became susceptible to infection as indicated by the GFP expression. Large differences were observed in the transfection efficiency as indicated by the percentage of hACE2-expressing cells which ranged from 5% (CpLu) to 50% (HypNi/1.1 and Tb 1 Lu). Among the hACE2-positive cells, about 10% were infected by pseudotypes containing the SARS-CoV S protein. The S protein of a porcine coronavirus, TGEV, was included in our analysis (Figure 2). Here, cells were not infected by pseudotypes but by the virus itself. Again, none of the bat cell lines was sensitive to infection. However, they became susceptible when pAPN was expressed on the cell surface. Infection was detected by staining for the presence of TGEV S protein. Interestingly, the staining pattern varied to a large extent depending on the cell line used. Bright staining distributed all over the cell was observed with HypNi/1.1 cells, while only a few fluorescent spots were detected in TGEV-infected EpoNi/22.1 cells expressing pAPN. This result shows that (i) TGEV infection of bat cells is restricted at the level of the cellular receptor, and (ii) there are large differences in the efficiency of the post-entry steps of the TGEV infection.

### Infection mediated by the S proteins of bat coronaviruses

**Figure 3:**
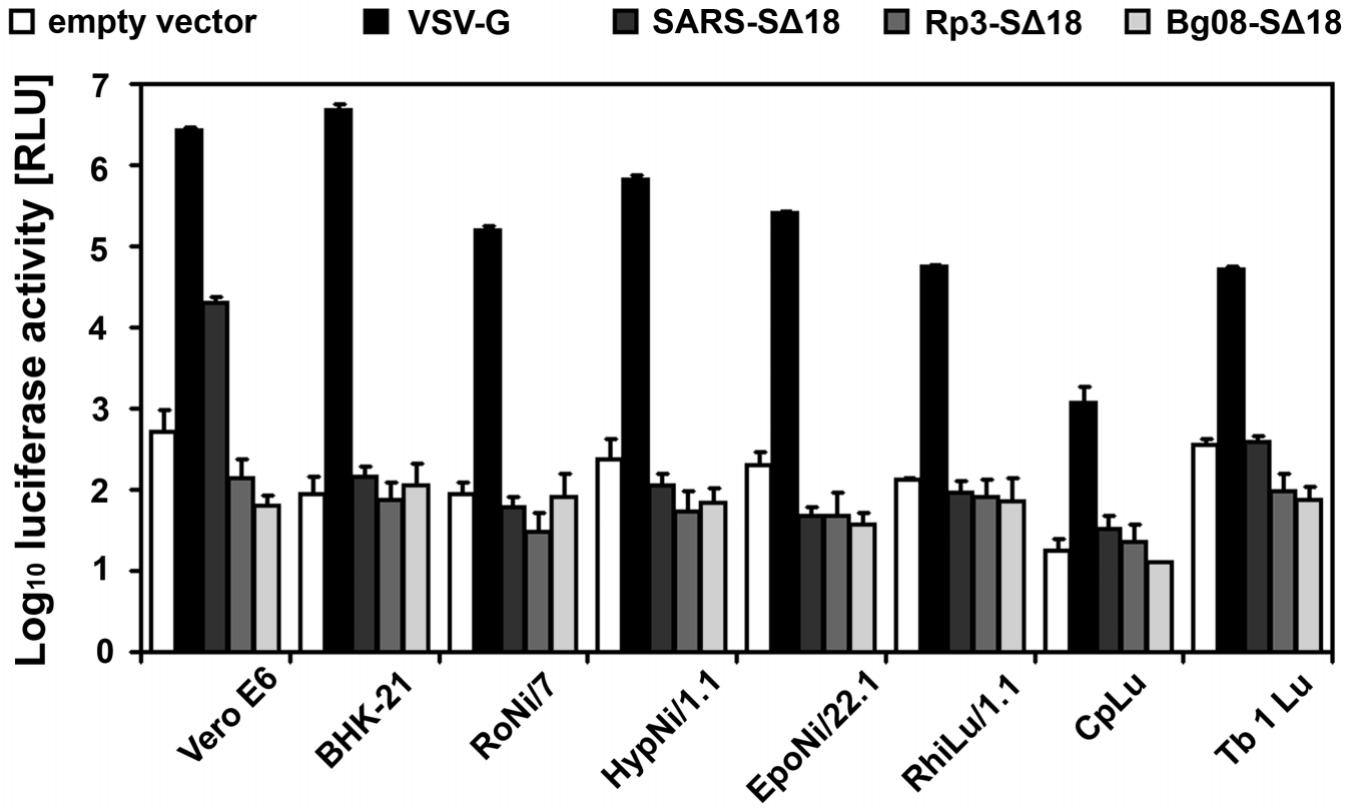
Susceptibility of bat cell lines to infection mediated by the S proteins of two bat-derived SARSr-CoVs, Rp3 and Bg08. VSV pseudotyped with either SARS-CoV SΔ18 (SARS SΔ18), SARSr-CoV Rp3 SΔ18 (Rp3 SΔ18), or SARSr-CoV Bg08 SΔ18 (Bg08 SΔ18) were applied to confluent bat cells and infection efficiency was determined by measuring the luciferase activity 18 h p.i.. VSV pseudotypes generated with VSV G (VSV G) or with an empty pCG1 vector alone (empty vector) served as positive and negative controls, respectively. All tests were performed in quadruplicates and the data shown are the result of three independent experiments.

Having shown that infection of bat cells by human and porcine coronaviruses is restricted at the entry stage, we wanted to know whether such restrictions are also observed when S proteins of bat coronaviruses are analyzed for the ability to mediate infection. As no replication-competent bat coronavirus is available up to now, we used the VSV pseudotype system to investigate whether the S proteins of the bat-derived SARSr-CoV Bg08 and Rp3 are able to infect any of the bat cells. The S proteins of these two viruses were highly distinct from each other (75% amino acid identity) and about equally distinct from the corresponding protein in SARS-CoV (SARSr-CoV Rp3 S: 79% vs. SARSr-CoV Bg08 S: 75% amino acid identity). It was shown previously, that the RBD of the European SARSr-CoV Bg08 is more related to that of SARS-CoV than that of the Chinese virus Rp3, which in turn is more related to SARS-CoV in most other genomic regions [9], [11]. In our comparative analysis, VSV G protein and the SARS-CoV S protein served as positive or negative controls, respectively. Pseudotypes containing the VSV G protein infected all cell lines, though at different efficiency (Figure 3). The low values determined in CpLu cells are due to the less efficient transfection and the slower growth of these cells. On the other hand, the S protein of SARS-CoV was only able to mediate infection of Vero E6 cells whereas in all bat cells only background signals were observed. The S proteins of Bg08 and Rp3 were also found to be unable to infect either of the bat cells (Figure 3).

### Infection mediated by the G protein of Marburg virus

**Figure 4:**
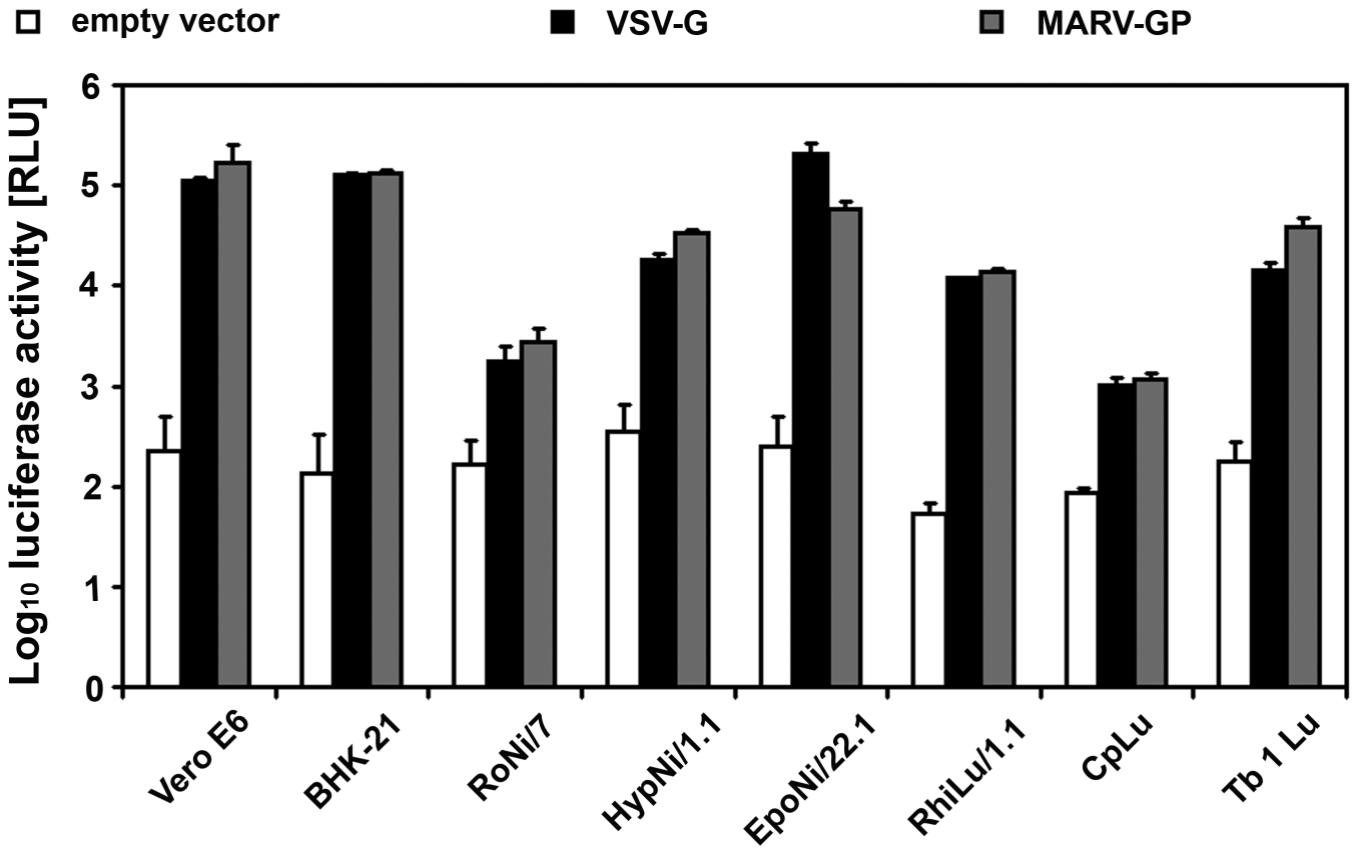
Sensitivity of bat cell lines to infection mediated by the GP of MARV. VSV pseudotypes containing VSV G (VSV G), MARV GP (MARV GP), or empty pCG1 vector alone (empty vector) were used to infect confluent bat cells. Infection was evaluated at 18 h p.i. by measuring the luciferase acticvity. Data shown are the result of three independent experiments.

A general restriction for virus entry can be ruled out as some of the applied bat cell lines (EpoNi/22.1 and HypNi/1.1) could be infected by VSV pseudotypes carrying Ebola virus glycoprotein [53]. As Marburg virus (MARV) was previously shown to be hosted by *Rousettus aegyptiacus* [48], [54] we extended the previous study by analyzing the surface protein GP of the related Marburg virus with a larger panel of bat cells. As shown in Figure 4, the filovirus glycoprotein mediated infection of all cells analyzed. Infection efficiency varied but this variation was also observed with the VSV G protein. In general, titres determined with the MARV GP were comparable to those obtained with the VSV G protein. Thus, in contrast to surface proteins of coronaviruses, the filovirus surface glycoprotein is able to infect bat cells in the context of a VSV pseudotype system.

### Interaction of coronaviral S proteins with human and chiropteran ACE2

**Figure 5:**
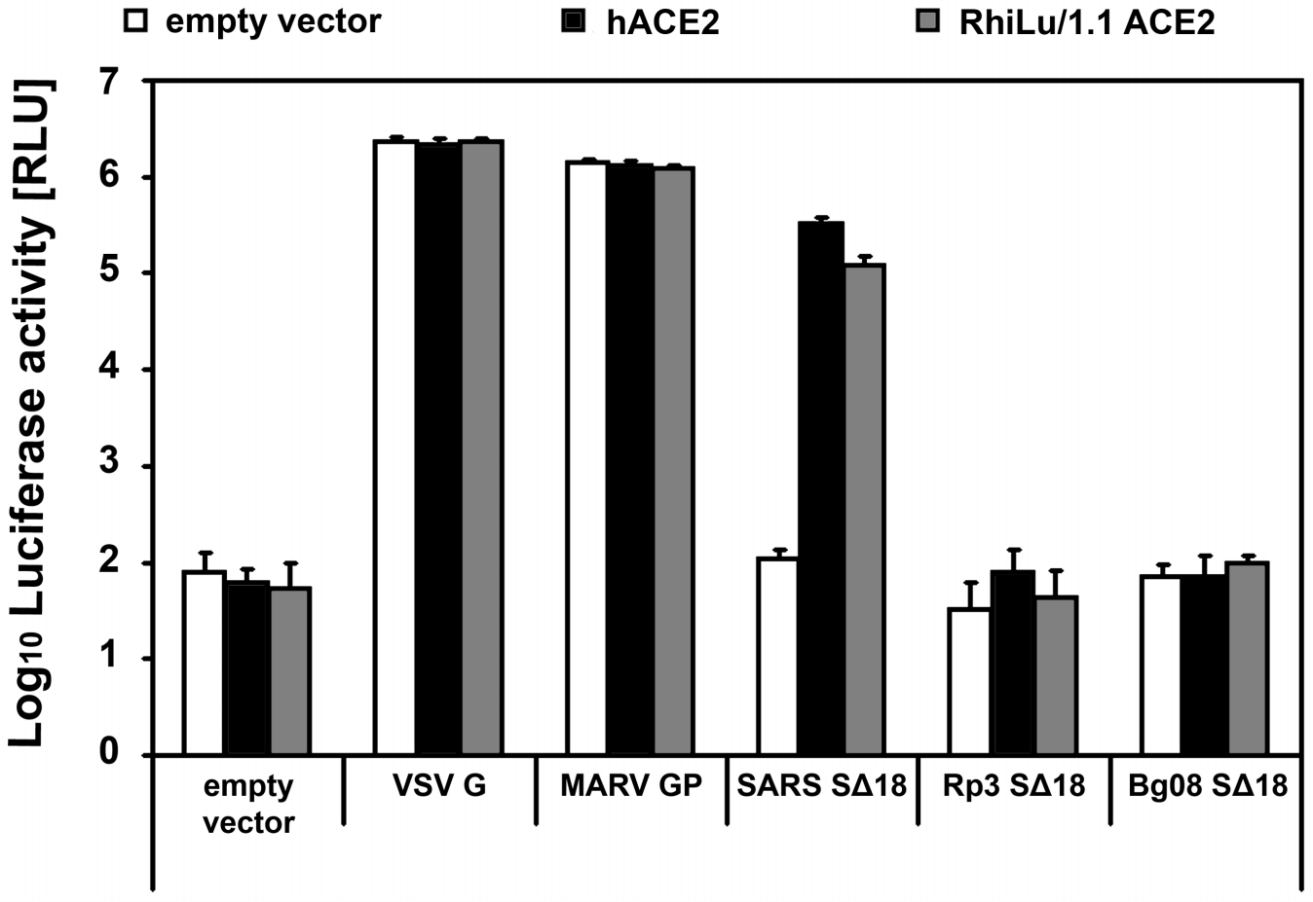
Analysis of the ability of human or RhiLu/1.1_ACE2 to serve as an entry receptor for VSV pseudotypes harboring SARS-CoV S, SARSr-CoV Rp3 S, or SARSr-CoV Bg08 S. BHK-21 cells, grown in white, opaque-walled 96well plates were transfected with expression plasmids for human (hACE2), *Rhinolophus alcyone* ACE2 (RhiLu/1.1_ACE2), or empty pCG1 vector alone (empty vector) prior to infection with VSV pseudotypes harboring either VSV G (VSV G), MARV GP (MARV GP), SARS-CoV SΔ18 (SARS SΔ18), SARSr-CoV Rp3 S Δ18 (Rp3 SΔ18), or SARSr-CoV Bg08 SΔ18 (Bg08 SΔ18). VSV pseudotypes generated with empty pCG1 vector alone (empty vector) served as a negative control. All tests were performed in quadruplicates and the data shown were obtained from three independent experiments.

After having shown that the inability of the SARS-CoV S and the TGEV S protein to mediate infection of chiropteran cells can be overcome when the respective receptor (hACE2 or pAPN, respectively) is expressed, we analysed whether expression of bat ACE2 renders cells susceptible to infection mediated by the S protein of coronaviruses. For this purpose, we compared the ability of VSVpp harboring the S proteins of SARS-CoV or either of the two SARSr-CoV, Bg08 and Rp3, to utilize human or rhinolophid ACE2 for initiating infection. The ACE2 coding sequence of the RhiLu/1.1 cell line, obtained from *Rhinolophus alcyone*, was analyzed for its ability to enable the entry of VSVpp. VSV pseudotyped with VSV G or MARV GP were able to infect BHK-21 cell, irrespective of ACE2 expression. SARS-CoV S mediated VSVpp infection of BHK-21 cells expressing hACE2. Interestingly, *Rhinolophus alcyone* ACE2 (RhiLu/1.1_ACE2) was very efficiently used for SARS-CoV S-driven pseudotype entry (Fig. 5). The infectivity mediated by RhiLu/1.1_ACE2 was almost as efficient as in the case of BHK-21 cells expressing hACE2. The S proteins of Bg08 and Rp3 were unable to mediate infection of cells expressing either hACE2 or bat ACE2.

**Figure 6:**
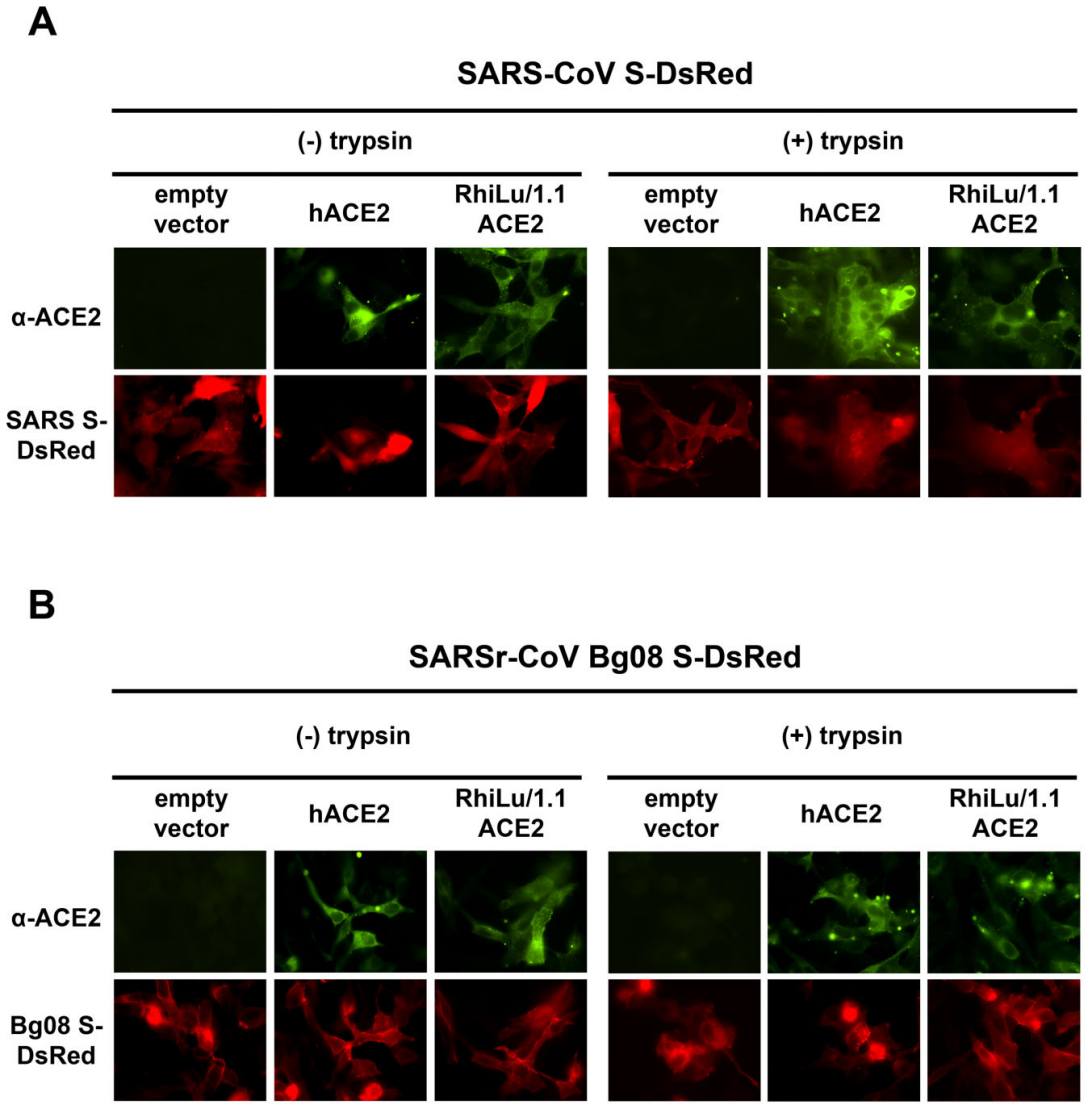
Effect of trypsin-treatment on the ability of SARS-CoV S and SARSr-CoV Bg08 S to induce syncytia formation when co-expressed with human or RhiLu/1.1_ACE2. BHK-21 cells, grown on coverslips were co-transfected with different combinations of expression plasmids for either (a) SARS-CoV S (SARS-CoV S-DsRed), or (b) SARSr-CoV Bg08 S (SARSr-CoV Bg08 S-DsRed), and ACE2 molecules of human (hACE2) or chiropteran (*Rhinolophus alcyone*, RhiLu/1.1_ACE2) origin. In both cases, empty pCG1 vector served as controls (pCG1). At 24 h post transfection, cells were either treated with medium containing trypsin (+ trypsin) to enable proteolytic activation of the CoV S or were left untreated (- trypsin). Subsequently, ACE2 was stained by antibody incubation (α-ACE2) and screened for syncytia formation by fluorescence microscopy. All tests were performed in triplicates and repeated three times.

To address the question whether the SARSr-CoV S protein is functional in a virus-free assay or can achieve functional activity after protease treatment, a finding that has been described for SARS-CoV S [33], [55]–[60], we performed a cell-based fusion assay, in which BHK-21 cells were co-transfected with combinations of expression plasmids for CoV S with a carboxyterminal DsRed-tag and different ACE2s. After transfection, cells were treated with trypsin. The presence of the two proteins was verified by fluorescence microscopy following immunostaining (ACE2). Trypsin-treated SARS-CoV S is able to induce fusion of the S-expressing cells with ACE2 expressing cells resulting in the formation of syncytia [55], [56]. We observed that SARS-CoV S was able to mediate fusion following trypsin-treatment, only with cells expressing hACE2 or RhiLu/1.1_ACE2 (Fig. 6a), as indicated by the detection for multinucleated cells that were positive for both, SARS-CoV S and the respective ACE2. In contrast, neither untreated nor trypsin-treated SARSr-CoV Bg08 S resulted in the formation of syncytia when co-expressed with either of the ACE2 proteins (Fig. 6b). Control experiments with cells expressing ACE2 proteins only did not reveal any syncytia formation (data not shown).

### Infection of bat cells by paramyxoviruses and influenza viruses

**Figure 7:**
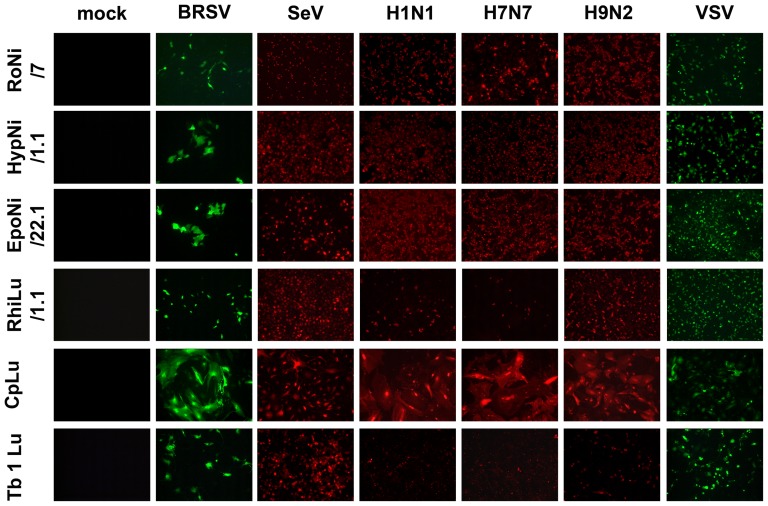
Sensitivity of bat cells to infection by paramyxoviruses and influenza viruses. Bat cells were grown on coverslips and subjected to infection by either BRSV(GFP) (BRSV), SeV(DsRed) (SeV), influenza A viruses of the subtypes H1N1 (H1N1), H7N7 (H7N7), and H9N2 (H9N2), as well as VSV(GFP) (VSV). Infection was monitored at 24 h or in case of BRSV(GFP) at 48 h p.i. via fluorescence microscopy either directly (BRSV(GFP), SeV(dsRed), VSV(GFP)) or after antibody staining of viral antigen (H1N1, H7N7, H9N2). In all cases, uninfected cells served as controls (mock). All tests were performed in triplicates and repeated three times.

Having shown that bat cells are susceptible to infection by VSV if an appropriate surface glycoprotein is incorporated into the viral envelope we analyzed whether other enveloped RNA viruses are able to infect bat cells. For this purpose we chose paramyxoviruses and influenza viruses. Recent data suggest that bats may also serve as a natural reservoir for paramyxoviruses [46]. As shown in Figure 7, the paramyxoviruses used in our study, bovine respiratory syncytial virus and Sendai virus, efficiently infected all six bat cell lines (RoNi/7, EpoNi/22.1, HypNi/1.1, RhiLu/1.1, CpLu, and Tb 1 Lu). Influenza viruses were also included in our study. A recent report demonstrated the presence of an influenza A virus in bats captured in Guatemala [37]. For our infection study with influenza viruses, we chose two low-pathogenic avian strains belonging to the subtypes H7N7 and H9N2 and a porcine H1N1 virus. These viruses differ in their recognition of sialic acid with the H7N7 virus having a preference for α2,3-linked sialic acid and the H1N1 virus showing preferential binding to α2,6-linked sialic acid. The H9N2 virus recognizes both linkage types. Despite the difference in the sialic acid binding activity, all three influenza viruses showed a similar infection pattern (Figure 7). RoNi/7, HypNi/1.1, EpoNi/22.1, and CpLu cells were highly sensitive to infection with almost all cells of the monolayer showing viral antigen. RhiLu/1.1 and Tb 1 Lu cells were also infected by influenza viruses, but less efficiently as indicated by the lower number of cells showing viral antigen. This result indicates that the general resistance of bat cells to infection by coronaviruses is not observed when influenza and paramyxoviruses are analyzed.

## Discussion

We have shown that the S proteins of two SARSr-CoV are unable to mediate infection of bat cells derived from different species of *Yinptero*- and *Yangochiroptera*. The resistance of the bat cells is not due to the pseudotype system used because (i) pseudotypes containing the VSV G protein or the G protein of Marburg virus were found to be quite efficient in infecting the different bat cells and (ii) in the case of the S protein of SARS-CoV, the resistance of the bat cells to infection by pseudotypes was overcome after the cells had been treated to express the receptor for SARS-CoV, hACE2. One might argue that the lack of infection in the case of the bat CoV S protein may be related to an inefficient particle production or to a functional impairment of the S protein due to the deletion of the carboxyterminal amino acids of the cytoplamic tail. This deletion was found to be favourable for the function of the S proteins of SARS-CoV and TGEV in VSV and lentiviral pseudotype systems [52], [61]–[63]. We do not know what is the effect of such a deletion in the case of the S proteins of bat CoV but the above experiments were also performed with native S proteins and the result was the same, i.e the S protein of SARS-CoV infected hACE2-expressing cells whereas the S proteins of the two bat SARSr-CoV were unable to mediate infection (not shown). As far as particle production is concerned, Western blot analysis of pseudotype particles in the cell supernatant indicated that the nucleoprotein N of VSV was present in similar amounts in pseudotype preparations irrespective of the source of the S protein (not shown). A receptor-dependent resistance to infection was also observed when the cells were infected with an infectious coronavirus, TGEV. Susceptibility of bat cells to infection by TGEV was dependent on the expression of porcine APN on the surface of the bat cells. These results suggest that the failure of the two S proteins of bat CoV to mediate infection in the context of VSV pseudotypes reflects a property of the viral glycoproteins rather than a problem of the pseudotype system. The inability of the Rp3 S protein to mediate the entry process has also been reported by others [15] and may be explained by a strict species specificity of bat coronaviruses. There are ecological studies suggesting host-restricted distribution of bat coronaviruses at the species level or at the genus level [3], [7], [11]. It is not known which step in the replication cycle is responsible for the host-restriction. If virus entry is the limiting step, our results would suggest that the S proteins interact only with receptors from cells of the same species but not of the same genus. The S proteins used were derived from *R. blasii* and *R. sinicus*, respectively. Cells of horseshoe bats, in our study, were derived from the species *R. alcyone* and they were resistant to S-mediated infection. An alternative explanation for the inability of the S proteins of bat coronaviruses to mediate infection may be the lack or insufficient expression of the cellular receptor on the surface of the bat cells. In this context it is interesting that, recently, SARS-CoV has been reported to use the ACE2 of different bats as a receptor for virus entry into Hela cells [64] which is confirmed by our results shown in Fig. 5. At present, it is not known whether bat coronaviruses use ACE2 or another protein as a receptor for infection. Immunostaining did not reveal detectable amounts of endogenous ACE2 expression in the bat cells analysed here. Transfected cells expressing ACE2 of RhiLu1.1 cells were also resistant to infection mediated by the S proteins of bat coronaviruses. As long as the identity of the cellular receptor for bat coronaviruses is not known, inefficient expression of this receptor would also explain why the two S proteins failed to mediate infection. This explanation may be applied also to other cellular factors required for virus entry, e.g post-adsorption (fusion) receptors or proteases required for induction of the fusion activity of the S proteins. The recently identified coronavirus MERS-CoV appears to differ from other human coronaviruses by a less restricted cell tropism [65]. It remains to be shown whether this property has arisen during transmission to human hosts and only applies to the virus of the diseased patients or whether it is also a characteristic of related bat viruses.

Our findings and the above mentioned explanations have important implications. They may explain why up to now all attempts to isolate infectious bat coronaviruses have failed [3], [4], [6], [7], [11], [13]–[17]. As a consequence, future attempts to isolate such viruses should consider that not all available bat cells are suitable host cells. Pseudotypes containing the S protein of bat coronaviruses should be helpful to identify cells susceptible to infection. Five of the cell lines used in this study were immortalized by SV40 large T antigen. CpLu cells were spontaneously immortalized. In the future, it may by worthwhile, to include primary cells in the analysis. As the respiratory and intestinal epithelium is a common target for many coronaviruses, it should be interesting to find out whether differentiated airway or intestinal epithelial cells are susceptible to S-mediated infection. This would indicate that the receptor is expressed upon differentiation of the cells. Alternatively, cell surface expression may be age-dependent which might be one explanation why genomic coronavirus RNA was detected more frequently in fecal samples from young animals than from adult bats [3].

The choice of cells to be used for isolation of bat coronaviruses is not only dependent on the presence of appropriate receptors. Intracellular factors may also be critical for a productive replication cycle. This is evident from the infection by TGEV. The expression of porcine APN was sufficient for virus entry, but the subsequent course of infection was quite different. In some cells viral antigen was distributed all over the cytoplasm, whereas in others expression of viral proteins was restricted to dot-like structures.

Infection of bat cells by filoviruses, paramyxoviruses, and influenza viruses appears not to be as restricted as infection by coronaviruses. This finding is interesting because for filoviruses and paramyxoviruses recent data suggest that bats may serve as a natural reservoir [46], [49]. Bats may also be a host for influenza viruses as indicated by a recent report where a virus strain – from bats captured in Central America – was identified and has been tentatively assigned to a new subtype, H17 [37]. Our results suggest that transmission of filoviruses, paramyxoviruses and influenza viruses from bats to new hosts appears to be less restricted at the level of cellular receptors than is transmission of coronaviruses. Infection of bat cells from different species by Ebola virus has been demonstrated [53] and our pseudotype experminents with the G protein of Marburg virus confirm and extend these results. Evidently, the receptor interaction of filoviruses is not species-specific. The reason for the different infection efficiency observed among the cells analyzed is not known. Future studies have to find out whether this difference is accounted for by a species-dependent recognition of the receptor or by the expression level of the cellular receptor for these viruses. In the case of influenza viruses and paramyxoviruses, infection was observed with all cells analyzed. For the different influenza viruses and for Sendai virus, successful infection can be explained by their use of sialic acid as a receptor determinant for infection of cells. Evidently, both α2,3-linked and α2,6-linked sialic acids are present on the surface of the cells.

Taken together, our results show that many bat cell lines are resistant to infection mediated by the S proteins of SARSr-CoV; viral pseudotypes can be used to identify susceptible cells and thus may help to isolate infectious SARSr-CoV from bats.

## Materials and Methods

### Cells and viruses

BHK-21 cells were maintained in Eagle's minimum essential medium (EMEM; Gibco) supplemented with 5% fetal calf serum (FCS; Biochrom) and 1% penicillin/streptomycin (PAA), Vero E6 and all bat cell lines were maintained in Dulbecco's minimal essential medium (DMEM; Gibco) supplemented with 10% FCS and 1% penicillin/streptomycin. The bat cell lines additionally were supplemented with 1 mM sodium pyruvate (PAA). All cells were cultivated in 75 cm^2^ tissue culture flasks (Greiner Bio-One) at 37°C and 5% CO_2_, and passaged 1∶4 (CpLu, Tb 1 Lu) or 1∶10 when they had reached 90% confluency. Bat cell lines from the following species were used in our studies: *Rousettus aegyptiacus* (kidney-derived; RoNi/7), *Hypsignathus monstrosus* (kidney-derived; HypNi/1.1), *Epomops buettikoferi* (kidney-derived; EpoNi/22.1), *Rhinolophus alcyone* (lung-derived; RhiLu/1.1, subcloned cell line), *Carollia perspicillata* (lung-derived; CpLu), and *Tadarida brasiliensis* (lung-derived; Tb 1 Lu; provided by Friedrich-Loeffler-Institut, Insel Riems, Germany). RoNi/7 (mixed cell culture), HypNi/1.1, EpoNi/22.1 (both subcloned cell lines) were generated from mechanically and enzymatically separated organ samples as described previously [53], [66], RhiLu/1.1, and CpLu cell lines were prepared accordingly. Apart from CpLu cells, all other bat cell lines were immortalized by the SV40 large T antigen [67]. RoNi/7, HypNi/1.1, EpoNi/22.1, and RhiLu/1.1 cell lines were generated from bats caught in Ghana. For all capturing, sacrificing, and sampling, permission was obtained from the Wildlife Division, Forestry Commission, Accra, Ghana. Samples were exported under a state contract between the Republic of Ghana and the Federal Republic of Germany, and under an additional export permission from the Veterinary Services of the Ghana Ministry of Food and Agriculture (permit no. CHRPE49/09; A04957). Under the auspices of Ghana authorities bats were caught with mist nets, anaesthetized with a Ketamine/Xylazine mixture and euthanized to perform organ preparations (permit no. CHRPE49/09; A04957). The CpLu cell line was generated from a *C. perspicillata* breeding colony maintained at the institute of zoology (University of Veterinary Medicine Hannover), for research purposes. The keeping and breeding of *Carollia perspicillata* was approved by the Landeshauptstadt Hannover, Fachbereich Recht und Ordnung, Gewerbe und Veterinärangelegenheiten (No. 42500/1H). The corresponding cell line is derived from postmortem tissue of bats sacrificed for other purposes. Animals were sacrificed (permit No. 11/0435) by cervical dislocation in deep inhalation anaesthesia (halothane).

In the present study we employed a vesicular stomatitis virus (VSV) pseudotype system that is based on a replication-deficient VSV replicon in which the open reading frame (ORF) of the VSV glycoprotein (VSV G) has been replaced by two individual ORFs for an enhanced green fluorescent protein (EGFP) and a firefly luciferase (Luc). This replicon (VSV*ΔG-Luc) has been constructed in collaboration with G. Zimmer [68] and has already been used in other studies [68]–[70].

Replication-competent viruses used for infection of bat cell lines were recombinant VSV that codes for an EGFP in an additional ORF between the VSV G and VSV L genes (VSV(GFP)), provided by G. Zimmer. Recombinant bovine respiratory syncytial virus that codes for an EGFP, BRSV(GFP), has been described recently [71]. Recombinant Sendai virus encoding dsRed, SeV(dsRed), has been described by Zimmer et al. [72]. The three influenza viruses used in this study comprise one porcine strain of the subtype H1N1 (A/swine/Potsdam/15/81) and two avian strains of the subtypes H7N7 (A/duck/Potsdam/15/80) and H9N2 (A/chicken/Saudi Arabia/CP7/98). The Purdue strain of TGEV was provided by Luis Enjuanes.

### Expression plasmids

The expression plasmid for the cellular receptor of SARS-CoV, human angiotensin converting enzyme 2 (ACE2, NM_021804.2, pCG1-hACE2) was constructed from the pcDNA3.1-hACE2 expression plasmid which was kindly provided by E. Snijder, and cloned into the pCG1 vector (kindly provided by R. Cattaneo) using *Xba*I and *Sal*I restriction sites. Additionally, we succesfully isolated and cloned the ACE2 coding sequence of the RhiLu/1.1 cell line, derived from *Rhinolophus alcyone* (as mentioned in the next section). The sequence will be submitted to GenBank. The coding sequence of the cellular receptor of TGEV, porcine aminopeptidase N (pAPN) was obtained by *reverse-transcription* (RT-) PCR of mRNA from ST (swine testicular) cells followed by primer-specific PCR. The PCR product was cloned into the pCG1 vector using *BamH*I and *Sal*I restriction sites. The sequence will be submitted to GenBank. The pcDNA3.1-VSV G as a positive control for VSV pseudotype preparation was provided by G. Zimmer; the pCAGGS-MARV GP construct was provided by S. Becker. For production of VSV pseudotypes harboring SARS-CoV and SARSr-CoV S we generated expression plasmids based on the pCG1 vector (pCG1-SARS CoV SΔ18, pCG1-SARSr-CoV Rp3 SΔ18, and pCG1-SARSr-CoV Bg08 SΔ18) using *BamH*I and *Xba*I restriction sites. In these CoV S proteins, the cytoplasmic tail regions were truncated by 18 amino acids, a feature that has been shown previously to result in an increased incoporation of CoV S into VSV pseudotypes [52]. The pCG1-SARS-CoV SΔ18, pCG1-SARSr-CoV Rp3 SΔ18 (from *Rhinolophus sinicus*) possess a tissue plasminogen activator signal sequence instead of the original signal peptide to promote CoV S cell surface expression [73], [74]. The pCG1-SARSr-CoV Bg08 SΔ18 was generated based on SARSr-CoV RNA that has been detected in fecal samples of a horseshoe bat (*Rhinolophus blasii*, BtCoV/BM48-31/Bulgaria/2008) from Bulgaria [11]. Additionally, DsRed-linked constructs of full-lenght SARS-CoV S (Frankfurt-1 isolate, AY291315.1, pCG1-SARS-CoV S-DsRed) and SARSr-CoV Bg08 S (pCG1-SARSr-CoV Bg08 S-DsRed) were generated for the cell-based fusion assay. Here, the respective CoV S was carboxyterminally fused to a non-flexible linker sequence (amino acid sequence: GPDPPVAT) and the coding sequence for a DsRed protein (EU827527.1).

### Cloning of chiropteran ACE2 from permanent bat cell lines

RhiLu/1.1 cells were used for RNA extraction using the RNeasy extraction kit (Qiagen) according to the manifecture's protocol. The concentration of the extracted RNA was quantified by an Eppendorf BioPhotometer Plus (Eppendorf) and directly used for RT-PCR using the SuperScript III First-Strand Synthesis System (Life Technologies). The synthesis of cDNA was performed following the instructions for an oligo dT-based RT-PCR as mentioned in the manifacture's protocol. Subsequently, 5 µl of the cDNA were used for an ACE2-specific PCR using the Phusion polymerase (Thermo Scientific). The primers for this PCR were designed on the basis of Hou *et al*. [64] (forward: CTTTCTAGAATGTCAGGCTCTTYCTGG/reverse: CCGGTCGACCTAAAABGAVGTCTGAACATCATC). The PCR was performed under the following conditions: 2 min at 98°C/40× (20 sec at 98°C/20 sec at 52°C/3 min at 72°C)/10 min at 72°C/storage until further use at 4°C. The success of the cloning was determined by gel electrophoresis. A band of the calculated size of ACE2 was obtained. Next, the DNA fragment was cloned into the pCG1 vector using the *BamH*I and *Sal*I restriction sites. To exclude mutations that were generated during the cloning process, at least 4 different clones were sequenced.

### Antibodies and reagents

Transfection of BHK-21 cells was performed using *Lipofectamine2000 reagent (Life Technologies), while* transfection of bat cell lines was carried out with the help of *Lipofectamine2000* LTX (*Life Technologies*) and addition of PLUS reagent (*Life Technologies*), both according to the manifacturer's protocol.

For detection of TGEV S and pAPN we used antibodies raised in mice (anti-S 6A.C3 kindly provided by L. Enjuanes; 1∶250; anti-pAPN G43 kindly provided by H. Laude; 1∶250). For the detection of ACE2 a polyclonal goat antibody (R&D Systems, 1∶250) was used. Infection of bat cell lines by influenza A viruses was analyzed using a monoclonal antibody directed against the viral nucleoprotein (NP) (Serotec; 1∶750). For fluorescence-labeling, Cy3-linked anti-mouse (Sigma Aldrich, 1∶750), Cy3-linked anti-goat (Sigma Aldrich, 1∶750), and FITC-labeled anti-goat (Sigma Aldrich, 1∶750) antibodies were used. All antibodies were diluted in phosphate buffered saline (PBS) containing 1% bovine serum albumin.

### Generation of replication-incompetent VSV pseudotypes

To investigate whether CoV S proteins are able to mediate infection of bat cells we used a VSV pseudotype system that has been described elsewhere [70] with slight modifications.

BHK-21 cells were seeded in Ø 10 cm cell culture dishes (Greiner Bio-One). As soon as they had reached 75% confluency they were transfected with 10 µg of pCG1-SARS-CoV SΔ18, pCG1-SARSr-CoV Rp3 SΔ18, pCG1-SARSr-CoV Bg08 SΔ18, pCG1-MARV GP, pcDNA3.1-VSV G (positive control), or pCG1 (negative control). At 16 h post transfection the supernatant was removed, cells were washed three times with PBS, and infected by VSV pseudotypes that were transcomplemented with VSV G, VSV*ΔG-Luc, at an MOI of 3 for 1 h. Subsequently, the inoculum was removed, cells were washed three times with PBS, and neutralization of residual input virus was carried out using a polyclonal anti-VSV serum raised in rabbit at a dilution of 1∶1,000. After incubation for 1 h at 37°C and 5% CO_2_, the neutralization medium was removed, cells were washed three times with PBS, and EMEM supplemented with 3% FCS was added. The VSVpp were harvested 16–20 h later, by collecting the supernatant followed by centrifugation (3.500× g) to remove cell debris, and kept at 4°C for up to one week.

### Susceptibility of bat cell lines to virus infection

The different bat cell lines were seeded in 24-well plates (Greiner Bio-One) containing coverslips (Roth, immunofluorescence analysis) or white, opaque-walled 96-well plates (Greiner Bio-One, luciferase assay) and grown to 75% confluency before they were subjected to infection. To investigate the importance of the surface expression of different ACE2s or pAPN for infection by VSVpp or TGEV, respectively, cells were grown to 50% confluency and transfection with either pCG1-hACE, pCG1-RhiLu/1.1_ACE2, pCG1-pAPN, or empty pCG1 vector (negative control) was performed 24 h prior to infection. Cells grown in 96well plates were transfected with 0.3 µg, while cells grown on coverslips where transfected with 1 µg of the respective expression plasmid.

For infection, the medium was removed by aspiration, cells were washed three times with PBS, and infected with 1×10^5^ ffu/ml of TGEV, BRSV(GFP), SeV(dsRed), H1N1, H7N7, H9N2, or VSV(GFP). Alternatively, cells were infected with undiluted VSVpp harboring either VSV G, MARV GP, SARS-CoV SΔ18, SARSr-CoV Rp3 SΔ18, SARSr-CoV Bg08 SΔ18, or no glycoprotein at all (pCG1, empty vector). After 1 h of incubation at 37°C and 5% CO_2_ under slight agitation, the inoculum was removed, the cells were washed three times with PBS and EMEM containing 2% FCS and 1% methylcellulose (Sigma Aldrich) was applied to reduce spread of the replication-competent viruses. In the case of the replication-deficient VSVpp, DMEM containing 2% FCS was used for further incubation.

### Detection of infection by immunofluorescence analysis (IFA) and fluorescence microscopy

At 24 h (in case of BRSV(GFP) at 48 h) post infection (p.i.), the cell culture supernatant was removed by aspiration and the cells were washed three times with PBS before they were fixed by incubation with 3% paraformaldehyde in PBS for 20 min at room temperature (RT). After fixation, the cells were washed three times with PBS and residual paraformaldehyde was quenched by incubation with 0.1 M glycine in PBS for 30 min at RT. For intracellular detection of TGEV S and influenza virus NP, cells were permeabilized with methanol/acetone (1∶1, v/v) for 30 sec at RT, followed by washing with PBS. Antibody incubation was carried out in humidity chambers with one drop of antibody solution between a strip of parafilm and the cells on the coverslip. After 1 h of incubation at RT, the coverslips were transferred back to 24-well plates, washed three times with PBS and incubated with a fluorescent dye-labeled secondary antibody under the same conditions. If the cells were transfected to express hACE2 or pAPN for studying the infection of VSVpp(SARS-CoV S Δ18) or TGEV, additional antibody staining was performed with antibodies directed against the respective receptor molecules under the same conditions. Subsequently, the cells were washed and the nuclei were stained by incubation with DAPI (Roth, 1 µg/ml ethanol) for 10 min at 37°C. Subsequently the cells were washed one time with PBS, 3 times with *aqua dest.*, and mounted with mowiol. Fluorencence microscopy was performed using the Nikon Eclipse Ti and the NIS Elements AR software (Nikon).

### Quantification of VSVpp infection

Quantification of the infection of bat cell lines by the different VSVpp was carried out (i) by observation of EGFP-positive cells under the fluorescence microscope or (ii) by determining the luciferase activity:

(i) VSVpp infected cells were fixed 18 h p.i. and representative fluorescence microscopical pictures were taken (data not shown). (ii) The cell culture supernatant was removed at 18 h p.i. by aspiration and the cells were lysed by incubation with luciferase cell culture lysis reagent (Promega) for 30 min at RT on an orbital shaker. Subsequently, freshly prepared luciferase substrate from the luciferase assay system (Promega) was added and the emitted light signal was measured after 1 min of incubation using a Chemiluminometer (TECAN).

### Cell-based fusion assay

BHK-21 cells were seeded in 24-well plates containing coverslips to reach 50% confluency the next day. Then, the cells were co-transfected with combinations of pCG1-SARS-CoV S-DsRed, pCG1-SARSr-CoV Bg08 S-DsRed, or empty pCG1 vector (negative control) and different ACE2s of human (pCG1-hACE2) or chiropteran (pCG1-RhiLu/1.1_ACE2) origin. At 24 h post transfection, the cells were washed 3 times with serum-free medium and either incubated with fusion medium (DMEM +2 µg/ml acetylated trypsin, Sigma Aldrich) or non-fusion medium (DMEM) for 4 h, before the cells were washed again three times and further incubated for 16–18 h with DMEM containing 3% FCS. At this point, the cells were fixed, permeabilized (as described before), and ACE2 expression was confirmed by antibody staining. The screening for the formation of syncytia was performed using the Nikon Eclipse Ti fluorescence microscope (Nikon) and representative pictures were taken.
